# The glycosaminoglycan-binding chemokine fragment CXCL9(74–103) reduces inflammation and tissue damage in mouse models of coronavirus infection

**DOI:** 10.3389/fimmu.2024.1378591

**Published:** 2024-04-15

**Authors:** Vivian Louise Soares Oliveira, Celso Martins Queiroz-Junior, Delphine Hoorelbeke, Felipe Rocha da Silva Santos, Ian de Meira Chaves, Mauro Martins Teixeira, Remo de Castro Russo, Paul Proost, Vivian Vasconcelos Costa, Sofie Struyf, Flávio Almeida Amaral

**Affiliations:** ^1^ Departamento de Bioquímica e Imunologia, Instituto de Ciências Biológicas, Universidade Federal de Minas Gerais, Belo Horizonte, Brazil; ^2^ Departament of Microbiology, Immunology and Transplantation, Rega Institute for Medical Research, KU Leuven, Leuven, Belgium; ^3^ Departamento de Morfologia, Instituto de Ciências Biológicas, Universidade Federal de Minas Gerais, Belo Horizonte, Brazil; ^4^ Departamento de Fisiologia e Biofísica, Instituto de Ciências Biológicas, Universidade Federal de Minas Gerais, Belo Horizonte, Brazil

**Keywords:** coronavirus, betacoronavirus, chemokine, glycosaminoglycan, inflammation, neutrophil

## Abstract

**Introduction:**

Pulmonary diseases represent a significant burden to patients and the healthcare system and are one of the leading causes of mortality worldwide. Particularly, the COVID-19 pandemic has had a profound global impact, affecting public health, economies, and daily life. While the peak of the crisis has subsided, the global number of reported COVID-19 cases remains significantly high, according to medical agencies around the world. Furthermore, despite the success of vaccines in reducing the number of deaths caused by severe acute respiratory syndrome coronavirus 2 (SARS-CoV-2), there remains a gap in the treatment of the disease, especially in addressing uncontrolled inflammation. The massive recruitment of leukocytes to lung tissue and alveoli is a hallmark factor in COVID-19, being essential for effectively responding to the pulmonary insult but also linked to inflammation and lung damage. In this context, mice models are a crucial tool, offering valuable insights into both the pathogenesis of the disease and potential therapeutic approaches.

**Methods:**

Here, we investigated the anti-inflammatory effect of the glycosaminoglycan (GAG)-binding chemokine fragment CXCL9(74-103), a molecule that potentially decreases neutrophil transmigration by competing with chemokines for GAG-binding sites, in two models of pneumonia caused by coronavirus infection.

**Results:**

In a murine model of betacoronavirus MHV-3 infection, the treatment with CXCL9(74-103) decreased the accumulation of total leukocytes, mainly neutrophils, to the alveolar space and improved several parameters of lung dysfunction 3 days after infection. Additionally, this treatment also reduced the lung damage. In the SARS-CoV-2 model in K18-hACE2-mice, CXCL9(74-103) significantly improved the clinical manifestations of the disease, reducing pulmonary damage and decreasing viral titers in the lungs.

**Discussion:**

These findings indicate that CXCL9(74-103) resulted in highly favorable outcomes in controlling pneumonia caused by coronavirus, as it effectively diminishes the clinical consequences of the infections and reduces both local and systemic inflammation.

## 1 Introduction

First reported in December 2019 and later declared a pandemic by WHO in March 2020, COVID-19 has over 700 million confirmed cases and nearly 7 million deaths globally (1). The causative agent of COVID-19, SARS-CoV-2, is a betacoronavirus characterized by positive single-stranded RNA, structured as a single linear RNA segment (2–4). Being highly contagious, SARS-CoV-2 is predominantly spread via different routes like aerosol, direct contact, and fecal-oral pathways, leading to the infection of the upper airways. With symptoms ranging from mild to severe, COVID-19 can lead to outcomes like pneumonia, acute respiratory distress syndrome (ARDS), systemic inflammation, multi-organ failure, and death (5). Similar to other coronaviruses, SARS-CoV-2 enters cells through the angiotensin-converting enzyme 2 (ACE2) receptor (6), inducing endocytosis and initiating an immune response (7).

While various treatment options, including antivirals (8, 9), corticosteroids (10, 11), and antimicrobials (12), have been explored, a comprehensive drug addressing all aspects of COVID-19 is yet to be approved. Despite the success of vaccines, there persists a need to comprehend disease pathogenesis and enhance treatment modalities in particular to control long-lasting inflammatory reactions (13–15). In this regard, mouse models play a pivotal role in advancing knowledge about the disease and screening potential drug candidates. Nevertheless, wild-type mice do not express ACE2, therefore exhibiting resistance to SARS-CoV-2, requiring the utilization of transgenic mice, as those expressing the human ACE-2 receptor (K18-hACE2) (16). Alternatively, other viruses such as murine hepatitis coronaviruses (MHV) can be viable alternatives for overcoming interspecies differences (17). MHV is a betacoronavirus from the Coronaviridae family, sharing RNA characteristics with viruses from the same family (18). Differently from the causative agent of COVID-19, MHV utilizes the CEACAM1a receptor for cell entry. Utilizing MHV mouse models of infection provides insights to understanding SARS-CoV-2 pathology, including comparable pathogenesis, the possibility of using different wild type or genetically modified mouse strains and, as a biosafety level 2 pathogen, to work in less restricted environments (19, 20). There are several strains of MHV, and for this study, MHV-3 was employed because it promptly induces significant but not lethal lung damage (20), allowing the proper evaluation of CXCL9(74–103) activity. Following intranasal inoculation, MHV-3 replicates in the respiratory epithelium, leading to a transient lung disease with functional pulmonary impairment. Secondary infections occur in organs like the liver and brain, and mice succumb from liver dysfunction, a consequence of MHV’s hepatotropism (20, 21).

The immune response against the Coronaviridae family involves a complex and intense inflammatory response in affected organs but also systemically (22–24). Amidst the myriad of inflammatory mediators associated with betacoronavirus pathology, chemokines play a crucial role in recruiting immune cells and shaping the overall immune response (25–27). Chemokines are small signaling proteins that guide leukocyte movement and are involved in inflammation, homeostasis, and angiogenesis (28). Based on the arrangement of N-terminal cysteine residues, chemokines can be classified into four subfamilies that are called CC, CXC, CX3C, and C chemokines. CXC chemokines, especially the ELR+ members like CXCL1, play a particular role in neutrophil recruitment (29). Other chemokines, such as CXCL10, play a crucial role in inflammation, particularly in the context of viral infections (30). Through its receptor, CXCR3, CXCL10 induces the recruitment and activation of leukocytes including activated T cells and natural killer cells, leading to systemic inflammation and tissue damage (31). Besides the direct effect of chemokines on cell chemotaxis, other molecules are involved in the recruitment of leukocytes *in vivo*, including the glycosaminoglycans (GAGs). GAGs are present on the cell surface and in the extracellular matrix, interacting with various proteins, including proteases, growth factors, cytokines, and chemokines and playing roles in cell recruitment, angiogenesis, tumor progression, embryogenesis, wound healing and homeostasis (32). Chemokines often have positive charges, allowing them to bind to negatively charged GAGs, such as heparan sulfate, heparin, and hyaluronic acid, manipulating the chemokine activity during inflammation (33–35). Notably, the interaction between chemokines and GAGs is critical for *in vivo* cell migration, establishing concentration gradients and leading to cell recruitment (36).

Chemokines and their receptors are key players in inflammation, making them targets for therapeutic interventions (37, 38). Inhibiting or enhancing chemokines and chemokine receptor expression or activity are important approaches for studying and finding new treatment options for inflammatory diseases mainly by controlling cell migration (39–41). The massive accumulation of activated leukocytes, including neutrophils, in the lungs signifies a deterioration in the clinical prognosis of individuals affected by COVID-19 (42). Currently, different studies have demonstrated that targeting the chemokine system and neutrophil migration and activation in models of betacoronavirus infection reduces the overall inflammatory response (43). Among different approaches to decrease chemokine-induced cell migration and excessive lung inflammation, modifying GAG-chemokine interaction can be an interesting anti-inflammatory strategy. Modified chemokines, including truncated forms and isoforms, are explored for therapeutic strategies, as they can engage in GAG binding without eliciting their effects through the chemokine receptors, thereby reducing chemokine activity (44). For instance, the COOH-terminal fragment of CXCL9, CXCL9(74-103), binds with high affinity to GAGs and competes with chemokines for GAG binding, reducing neutrophil recruitment and inflammation in different animal models (45–48). In an experimental model of pneumonia caused by *Klebsiella pneumoniae*, the treatment with CXCL9(74-103) reduced neutrophil accumulation into the lung airways and the production of IL-1β without affecting bacterial control (46). In this context, we aimed to understand whether and how a GAG-binding peptide can affect the inflammation and the pathogenesis of murine viral infections like COVID-19, leading to important conclusions about the disease and helping in the development of new anti-inflammatory therapeutic options.

## 2 Materials and methods

### 2.1 Mice and reagents

For the MHV-3 experiments, six to eight weeks old, male C57BL/6 were acquired from the Central Animal House of UFMG and kept in the animal facility that belongs to the Biochemistry and Immunology Department at UFMG, registered in the CTNBio. For the SARS-CoV-2 experiments, ten to twelve weeks old, male and female transgenic mice expressing human ACE-2 receptor (K18-hACE2- mice, *Mus musculus*) were acquired from Jackson Laboratories and the experiments were performed in the Animal Biosafety Level 3 (BSL-3) multiuser facility from the Institute of Biological Sciences at UFMG. All the mice were maintained in a controlled environment, with ad libitum food and water at 29-30 °C, in a 12-h dark-light cycle and humidity of 50–58%. Experiments were performed according to the animal welfare guidelines of the Brazilian Guideline for the Care and Use of Animals in Teaching or Scientific Research Activities. They were approved by the Animal Ethics Committees of UFMG (License 420/2018 and 005/2021).

The MHV-3 strain was provided and sequenced by Clarice Weis Arns and Ricardo Durães-Carvalho from the Universidade Estadual de Campinas (UNICAMP, Brazil), and propagated in L929 cells. The SARS-CoV-2 gamma variant (also known as P1 lineage; #EPI_ISL_1060902, hCoV-19/Brazil/AM-L70-71-CD1739/2020) was isolated on Vero E6 cells from nasopharyngeal swabs of confirmed cases. All procedures related to SARS-CoV-2 culture were executed at a biosafety level 3 (BSL3) multiuser facility, while all the procedures related to MHV-3 culture were executed at a biosafety level 2 (BSL2) laboratory, according to WHO guidelines (49). Virus titers were determined as plaque forming units (PFU)/mL and virus stocks were kept in -80°C ultralow freezers.

The CXCL9(74-103) COOH-terminal peptide was chemically synthesized using fluorenyl methoxycarbonyl (Fmoc) chemistry using an Activo-P11 automated synthesizer (Activotec, Cambridge, UK), as previously described by Loos et al. (50). After synthesis, the peptides were dissolved in 0.1% trifluoroacetic acid (TFA – Sigma-Aldrich, Burlington, Massachusetts, USA) and purified by RP-HPLC. Peptides were loaded on a 150×10 mm Proto 300 C18 column (Higgins Analytical Inc., Mountain View, CA, USA) in 0.1% TFA in water at a flow rate of 4 mL/min and eluted in an acetonitrile gradient in water containing 0.1% TFA. Eluted proteins were detected by splitting 0.7% of the volume of the column effluent to an ion trap mass spectrometer (Amazon SL, Bruker, Bremen, Germany). For the experiments, mice received 100 µg of CXCL9(74-103) in 0.9% saline intravenously every 12 h. This treatment scheme was previously standardized by Vanheule, et al. (48) and also considers the *in vivo* stability and biodistribution of the CXCL9(74-103) peptide in other models (46, 48, 51).

### 2.2 Animal models

#### 2.2.1 MHV-3 model

Mice were anesthetized with a solution of ketamine (80 mg/kg – Syntec, Tamboré, São Paulo, Brazil) and xylazine (15 mg/kg – Syntec), subcutaneously. For the induction of viral pneumonia, mice were inoculated intranasally with 10^3^ PFU of MHV-3 in 30 µL of 0.9% sterile saline (Equiplex, São Paulo, São Paulo, Brazil) (20). All the animals in the control group received the same volume of the vehicle (0.9% sterile saline solution) by the same route. Mice were monitored daily for three days for body weight analysis. In the case of weight loss higher than 25%, euthanasia was performed to alleviate animal suffering. Three days after the challenge, euthanasia was performed using an overdose of anesthetic (ketamine and xylazine). Bronchial alveolar lavage fluid (BALF) was obtained by the instillation of 500 μL of phosphate buffered saline (PBS – Invitrogen, Waltham, Massachusetts, USA) through a catheter in the trachea. The fluid was withdrawn and instilled again two more times, PBS instillation was repeated three times, and the lavages were pooled. After perfusion, lungs were collected for viral titration and histopathological analysis. The BALF was centrifuged (5 min, 300 × g, 4 °C) and the supernatant was collected for the analysis of protein levels by Bradford assay. Furthermore, part of the resuspended cell pellet was used for cell counting.

#### 2.2.2 Sars-CoV-2 and K18-hACE2-mice model

Mice were subcutaneously anesthetized with a solution of ketamine (60 mg/kg) and xylazine (4 mg/kg) and inoculated intranasally with 10^5^ PFU of SARS-CoV-2 gamma strain in 10 µL of Dulbecco’s Modified Eagle’s Medium high glucose (DMEM – Cultilab, Campinas, São Paulo, Brazil) (52, 53). All the animals in the control group received the same volume of the vehicle (DMEM high glucose) by the same route. The animals were monitored daily for five days for body weight analysis. In the case of weight loss higher than 25%, euthanasia was performed to alleviate animal suffering. Five days after the challenge, the clinical score was determined based on the fur aspect (0 to 2 points), back arching (0 to 2 points), level of activity (0 to 2 points) and body weight loss (0 to 5 points). Euthanasia was performed using an overdose of anesthetic (ketamine and xylazine) and the lungs were perfused with 20 mL of saline solution to remove the circulating blood. Lungs were then collected, pottered, and homogenized in 500 µL of a phosphatase and protease inhibitor cocktail (Complete, mini EDTA-free Roche Applied Science, Mannheim, Germany) for 30 s using an Ultra-Turrax Disperser T-10 basic IKA (Guangzhou, China) for virus titration and RNA quantification. Additionally, lungs were preserved for histology.

### 2.3 Virus titration

To determine the viral load in the MHV-3 experiments, a plaque assay was performed. Serial dilutions of tissue homogenates were added onto a confluent monolayer of L929 cells in 24-well plates. Plates were incubated for 1 h at 37 °C and were gently agitated every 10 min to assure equal distribution of the sample. Subsequently, cultures were covered with the overlay medium (DMEM) containing 0.8% carboxymethylcellulose (CMC – Sigma-Aldrich), 2% fetal calf serum (FCS - Cultilab). Plates were incubated for 2 days, at 37 °C, and 5% CO_2_. After incubation, cultures were fixed with 10% neutral buffered formaldehyde (LabSynth, Diadema, São Paulo, Brazil) for 1 h and stained with 0.1% crystal violet (Laborclin, Curitiba, Paraná, Brazil). Virus titers were determined by visual analysis of the plaques and expressed as plaque-forming units (PFU). The reader was blinded to the source of the supernatant.

### 2.4 qPCR

Following dissection, small lungs were removed from the mice and stored on dry ice until further use. Using the TRIzol™ Reagent (Invitrogen), the lungs were subjected to homogenization and RNA extraction according to the manufacturer’s instructions. Subsequently, the RNA was converted to cDNA using High-Capacity RNA-to-DNA^TM^ kit (Applied Biosystems, Thermo Fisher Scientific, Waltham, Massachusetts, USA). Integrated DNA Technologies primers were used to analyze the gene expression of CXCL10 (Mm.78626005). 18S (Mm.414116100) was used as the housekeeping gene. Per reaction, 10 ng cDNA was used. qPCR was performed using the PowerTrack™ SYBR Green Master Mix (Applied Biosystems) and the 7500 Real-Time PCR system (Applied Biosystems). Relative gene expression was determined using the 2^-ΔΔCt^ method.

Viral RNA extraction was conducted using the QIAamp Viral RNA kit (Qiagen, Venlo, The Netherlands) following the manufacturer’s instructions. Quantitative RT-PCR assays were performed using the GoTaq^®^ Probe qPCR and RT-qPCR Systems (Promega, Madison, Wisconsin, USA) on a StepOne™ Real-Time PCR System (Applied Biosystems) and the ABI PRISM 7500 Sequence Detection System (Applied Biosystems). Amplifications were carried out in 25 µL reaction mixtures, including 2× reaction mix buffer, 50 µM of each primer, 10 µM of probe, and 5 µL of RNA template. The primer, probe, and cycling conditions recommended by the Centers for Disease Control and Prevention (CDC) protocol were employed for the detection of SARS-CoV-2.

Virus quantification utilized the standard curve method. For normalization to the cell amounts used, the housekeeping gene RNAse P was amplified, and Ct values for this target were compared with those obtained for different cell amounts (ranging from 10^7^ to 10^2^) for calibration. Alternatively, genomic (ORF1) and subgenomic (ORFE) regions were detected using specific primers and probes, as described by Wölfel et al. (54).

### 2.5 Blood leukocytes

The number of circulating blood leukocytes was determined in blood samples using the Celltac MEK-6500K hemocytometer (Nihon Kohden, Tokyo, Japan). For that, blood was collected from the inferior vena cava with EDTA (Bioclin, Belo Horizonte, Minas Gerais, Brazil) coated syringes and immediately analyzed.

### 2.6 BALF protein measurement

To assess the edema formation and the extent of the tissue damage, the concentration of protein in the BALF was measured using Bradford assay (Bio-Rad, Hercules, California, USA). Briefly, the working reagent is diluted 5 times and mixed with the BSA standards and samples. After an incubation of 30 min at RT, the absorbance was measured at a wavelength of 595 nm (800 TS Absorbance Reader with the Gen5 software – both from Biotek).

### 2.7 Pulmonary function test in mouse model of MHV-3 infection

Invasive forced spirometry was performed to evaluate lung function. As previously described by Russo et al. (55), mice were anesthetized with ketamine and xylazine, tracheostomized, placed in a body plethysmograph, and connected to a computer-controlled ventilator (Forced Pulmonary Maneuver System; Buxco Research Systems, Wilmington, NC, USA). Under mechanical respiration, the tidal volume (TV), volume per minute (MV), peak of compliance (Cpk), dynamic compliance (Cdyn), and lung resistance (Rl) were determined by the resistance and compliance test. Next, a quasistatic Pressure-Volume maneuver was performed to obtain the total lung capacity (TLC), residual volume (RV), and inspiratory capacity (IC). This maneuver consists in inflating the lungs to +30 cm of H_2_O and slowly exhaling until -30 cm of H_2_O. Then, the lungs were inflated to +30 cm of H_2_O and immediately connected to a highly negative pressure to enforce expiration till -30 cm of H_2_O, to identify the fast-flow volume. The forced vital capacity (FVC) and forced expiratory volume at 20 or 50 ms (FEV 20 or FEV50) were measured during this last maneuver, and the Tiffeneau–Pinelli index (FEV20/FVC or FEV50/FVC) was calculated using these two variables. Suboptimal maneuvers were rejected, and for each test in every single mouse, at least three acceptable maneuvers were conducted to obtain a reliable mean for all numeric parameters.

### 2.8 Lung histology

The collected lungs were fixed overnight with formaldehyde (4% in PBS), dehydrated, and embedded in paraffin to obtain tissue slices with 5 µm thickness using a microtome. The slices were fixed and stained with hematoxylin and eosin (H&E, Sigma-Aldrich) for microphotograph analysis. The tissue morphological alterations observed in the lungs were determined using an inflammatory score system: (I) airway inflammation (0 to 4 points), (II) vascular inflammation (0 to 4 points), (III) parenchymal inflammation (0 to 5 points), and (IV) polymorphonuclear cell infiltration (0 to 5 points) (56). The histological analysis was performed by an independent pathologist that was blinded to the experimental conditions.

### 2.9 Statistical analysis

The data were analyzed using the GraphPad PRISM software (GraphPad, USA, version 9.0.0). One-way ANOVA test followed by Bonferroni correction was used in the graphs with normal distribution. Otherwise, Kruskal-Wallis with Dunn’s multiple comparisons test was used. Pearson correlation was performed when appropriate. Significance was determined by comparing the different treated groups with the mock, unchallenged group, and the non-treated, vehicle, group. P-values were indicated as follows: * = p< 0.05 when compared with the corresponding mock group and # = p<0.05 when comparing treated groups and vehicle.

## 3 Results

### 3.1 Administration of CXCL9(74-103) improves various inflammatory indicators during MHV-3 infection

**Figure 1:**
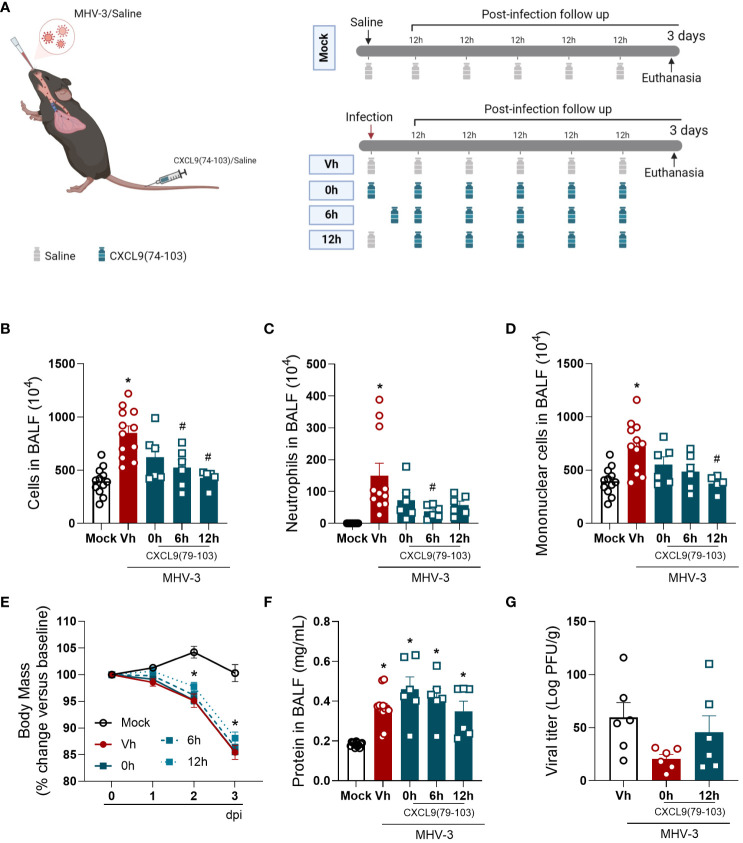
CXCL9(74-103) treatment improves several inflammatory parameters in MHV-3 infection. **(A)** C57BL/6 mice were intranasally infected with MHV-3 (3×10^3^ PFU/mouse) or Saline (Mock group) and dissected 3 days later. Starting from the indicated times after the challenge, mice were intravenously treated twice daily with CXCL9(74-103). Vehicle group (Vh) was infected with MHV-3 and treated twice daily with 0.9% saline solution. **(B)** After the euthanasia, BALF was collected, centrifuged and numbers of leukocytes were counted in the Neubauer chamber. Numbers of neutrophils **(C)** or mononuclear cells **(D)** in BALF were counted on cytospin slides. Mice were monitored daily and changes in body weight **(E)** were calculated with the weight before infection (day 0) as reference. The concentration of protein in BALF was measured by Bradford assay to assess the pulmonary edema **(F)**. Viral load in the lungs was calculated by titration **(G)**. Data are shown as mean ± SEM. Each symbol in panels **(B–D, F)**, and **(G)** represents data of an individual mouse. *p< 0.05 when compared with the healthy, unchallenged mock group. #p<0.05 when comparing different time points of treatment start (0h, 6h, or 12h) with vehicle treated mice. n=6-12. **Figure 1A** was created with BioRender.com.

To start the investigation of the impact of CXCL9(74-103), we used the MHV-3 model (**Figure 1A**), a model that emulates severe respiratory distress syndrome in mice (20). In this model, infected mice showed an increase in the number of cells, especially neutrophils and mononuclear cells, present in the BALF three days post-challenge, as illustrated in **Figure 1B–D**, respectively. In addition, on days 2 and 3 post-infection, a reduction in body weight was observed among the infected mice (**Figure 1E**). At the later time point, challenged mice had elevated protein levels in the BALF (**Figure 1F**) and a significant viral load in the lung tissue (**Figure 1G**). We initiated the treatments with CXCL9(74-103) at different time points to evaluate how this peptide could modulate cell recruitment and inflammation according to the temporal progression of the inflammatory response. Overall, the administration of CXCL9(74-103) reduced the number of total cells, neutrophils, and mononuclear cells recruited to alveolar space, with certain statistical variation among the treated groups. Even with the decrease in leukocytes, there was no observed difference in the viral load within the lungs after treatment. Furthermore, the peptide did not affect the leakage of protein into the BALF or the reduction in body weight in all treated groups.

### 3.2 The administration of CXCL9(74-103) improves lung function and tissue damage during MHV-3 infection

**Figure 2:**
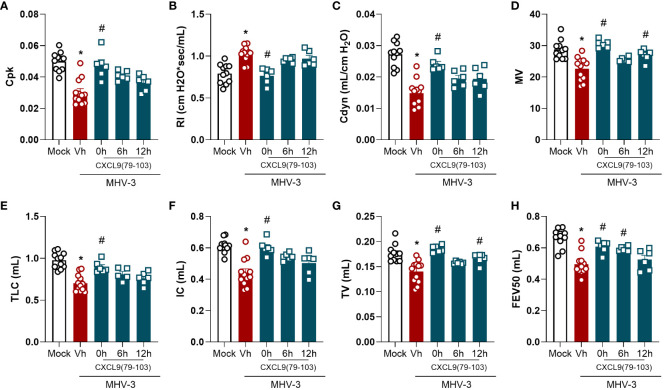
CXCL9(74-103) treatment improves several parameters of lung function in MHV-3 infection. C57BL/6 mice were intranasally infected with MHV-3 (3×10^3^ PFU/mouse) or Saline (Mock group) and dissected 3 days later. Starting from the indicated times after the challenge, mice were intravenously treated twice daily with CXCL9(74-103). Vehicle group (Vh) was infected with MHV-3 and treated twice daily with 0.9% saline solution. Right before the euthanasia, pulmonary mechanic functions were assessed. Invasive forced spirometry was performed to investigate functional modifications in pulmonary mechanics. The assessed parameters were Lung elasticity represented by **(A)** Peak of Compliance (Cpk), **(B)** Lung Resistance (RI), and **(C)** Dynamic Compliance Forced (Cdyn); **(D)** Lung volumes by Minute Volume (MV), **(E)** Total Lung Capacity (TLC), **(F)** Inspiratory Capacity (IC), and **(G)** Tidal Volume (TV); **(H)** Airway flow by Forced Expiratory Volume at 50 ms (FEV50). Data are shown as mean ± SEM. Each symbol represents data of an individual mouse *p< 0.05 when comparing with the healthy, unchallenged mock group. #p<0.05 when comparing different time points of treatment start (0h, 6h, or 12h) with vehicle treated mice. n=6-12.

Given the impact of CXCL9(74-103) on lung inflammation, we then evaluated the pulmonary function of MHV-3-infected mice treated or not with the CXCL9 terminal peptide. As observed in **Figure 2**, the MHV-3 infection markedly altered all the analyzed pulmonary mechanical parameters, reducing Lung elasticity depicted by Peak of Compliance (Cpk – **Figure 2A**), Dynamic Compliance Forced (Cdyn – **Figure 2C**), Lung volumes by Minute Volume (MV – **Figure 2D**), Total Lung Capacity (TLC – **Figure 2E**), Inspiratory Capacity (IC – **Figure 2F**), and Tidal Volume (TV – **Figure 2G**), Airway flow by Forced Expiratory Volume at 50 ms (FEV50 – **Figure 2H**), while increasing Lung Resistance (RI - **Figure 2B**) when compared to the mock infected group. Remarkably, the pretreatment (0h group) with CXCL9(74-103) effectively restored these parameters to basal levels, preventing the pulmonary mechanical distress induced by MHV-3. On the other hand, when the treatment started 6 h or 12 h after the infection, there was no improvement in every parameter, only a better outcome in MV, TV, and FEV50 (**Figures 2D, G, H**).

**Figure 3:**
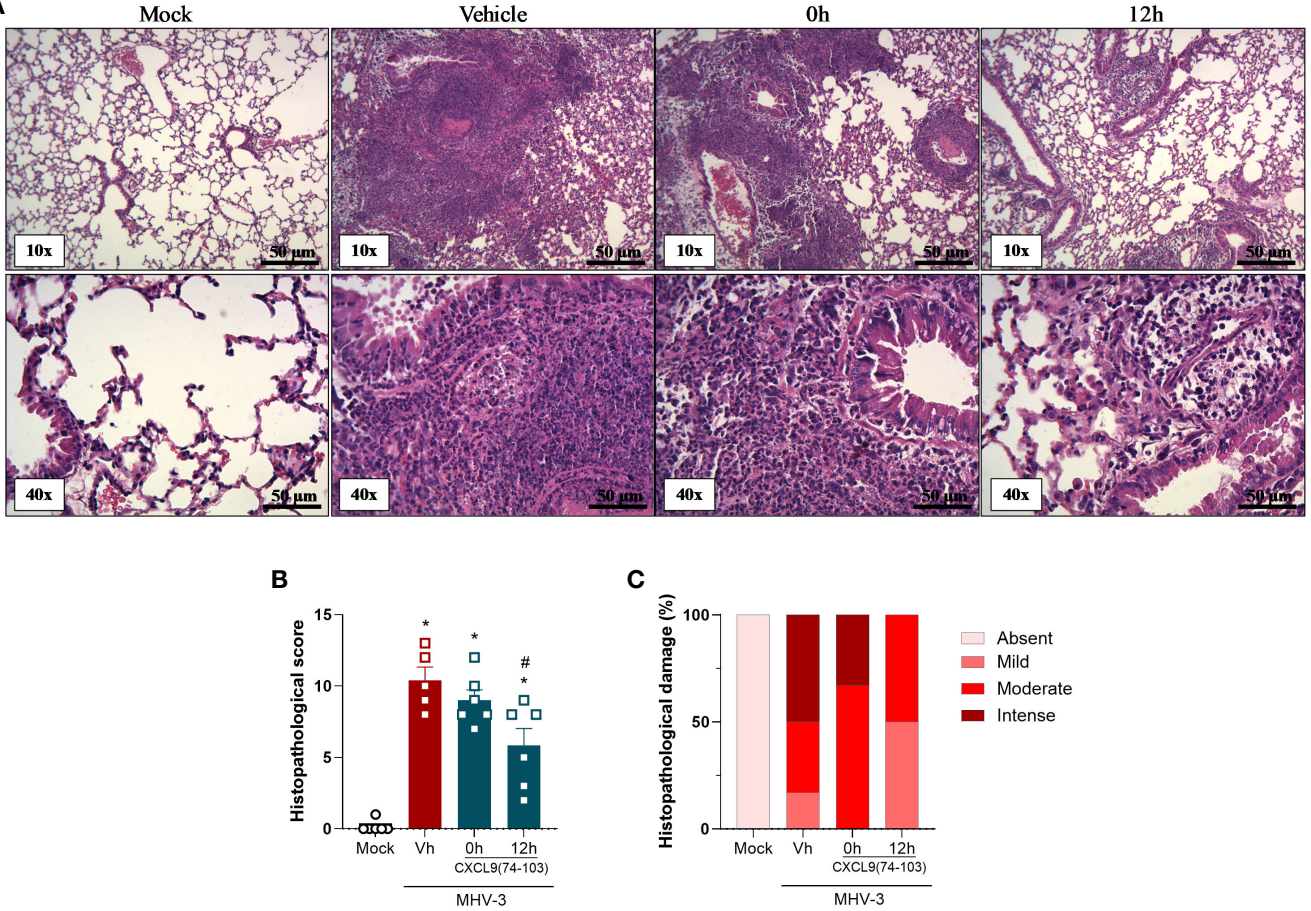
CXCL9(74-103) treatment improves tissue damage in MHV-3 infection. C57BL/6 mice were intranasally infected with MHV-3 (3×10^3^ PFU/mouse) or Saline (Mock group) and dissected 3 days later. Starting from the indicated times after the challenge, mice were intravenously treated twice daily with CXCL9(74-103). Vehicle group (Vh) was infected with MHV-3 and treated twice daily with 0.9% saline solution. **(A)** Representative hematoxylin and eosin-stained preparations of lung tissue from mice. Scale bar: 50 μm, as reported in the figure. **(B)** Histopathological score and **(C)** Contingency graph according with ranges of tissue damage (severe, intense, moderate, mild, and absent). Data are shown as mean ± SEM in panel **(B)**. *p< 0.05 when compared with the healthy, unchallenged mock group. n=5-6.

Lastly, tissue damage was evaluated by histopathological analysis. As observed in **Figure 3**, the MHV-3 infection caused a massive influx of leukocytes and the destruction of the airway walls, which can be directly related to the forced spirometry results. Nevertheless, in contrast to lung function measurements, mice treated 12 h after the challenge displayed a significant improvement in the histopathological score and damage (**Figures 3B, C**), showing that this group has less intense and frequent tissue damage, while the 0 h group did not have significant improvement when compared with the vehicle group. It is important to notice that only the 0 h and 12 h groups were selected for the histopathological analysis, given that, the overall results of the 6 h group were not superior to those of the 12 h group.

Altogether, the treatment with this GAG-binding peptide, especially when administered prior to the infection, was able to reduce the inflammation caused by MHV-3 by decreasing the number of total cells and neutrophils in the BALF and the viral titer in the lungs (**Figure 1**). Furthermore, CXCL9(74-103) improved the lung dysfunction (**Figure 2**) and the tissue damage (**Figure 3**).

### 3.3 CXCL9(74-103) treatment markedly improves the pulmonary pathology induced by SARS-CoV-2 infection

**Figure 4:**
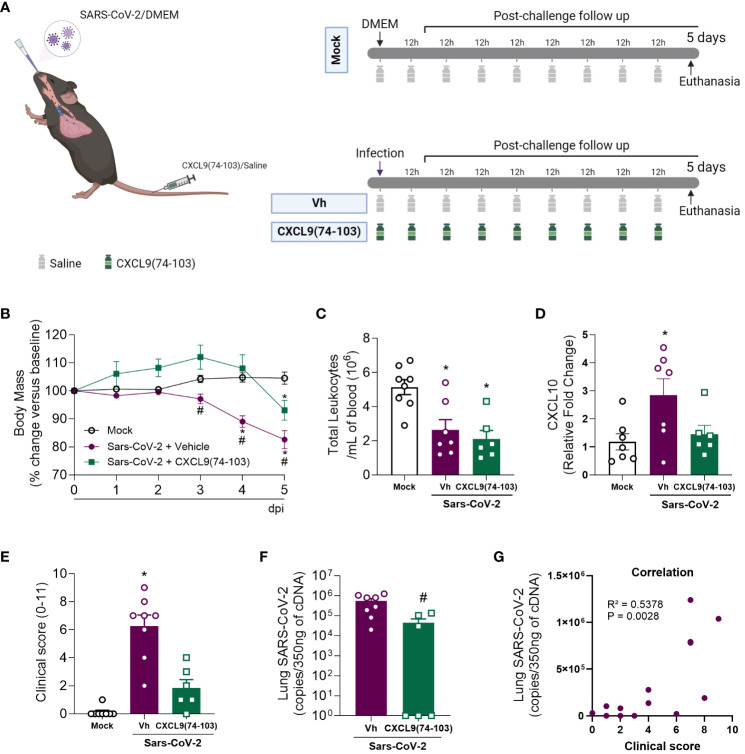
CXCL9(74-103) treatment prevents the increase in CXCL10 expression and reduces clinical parameters of disease and virus replication in Sars-Cov-2 infection. **(A)** K18-hACE2-mice were intranasally infected with SARS-CoV-2 (10^5^ PFU/mouse) or DMEM (Mock group) and dissected 5 days later. Mice were intravenously treated twice daily with CXCL9(74-103) since day 0. Vehicle group (Vh) was infected with SARS-CoV-2 and treated twice daily with 0.9% saline solution. Mice were monitored daily and changes in body weight **(B)** were calculated with the weight before infection (day 0) as reference. **(C)** Total blood leukocytes were measured by an automated cell counter. **(D)** Expression of CXCL10 in the lung tissue was determined by qPCR. **(E)** Mice were evaluated regarding the disease severity and the clinical score was determined. Viral load was calculated in the lungs by qPCR **(F)** and the correlation between the number of viral copies in the lungs and the severity of the disease expressed by the clinical score was determined **(G)**. Data are shown as mean ± SEM. Each symbol in panels **(C–F)** represents data of an individual mouse. *p< 0.05 when compared with the healthy, unchallenged mock group. #p<0.05 when comparing the treated group with vehicle (Vh). For panel **(G)**, Pearson correlation was used. n=6-8. **Figure 4A** was created with BioRender.com.

After the promising results observed in the MHV-3 model, we transitioned to a more clinically relevant model closely similar to human COVID-19. For this purpose, we used the SARS-CoV-2 virus and transgenic mice expressing the human ACE-2 receptor (K18-hACE2 mice). In K18-hACE2 mice infected with SARS-CoV-2, there was a reduction in body weight (**Figure 4B**), and in the blood leukocyte count (**Figure 4C**) five days after the challenge when compared with the mock group. Simultaneously, an increase in CXCL10 expression was detected in the lung tissue of the infected mice (**Figure 4D**), suggesting enhanced inflammation in response to the virus. Furthermore, the challenged mice had exacerbated clinical manifestations of the disease, as indicated by the clinical score (**Figure 4E**), and a considerable viral load in the lung tissue (**Figure 4F**). Remarkably, a positive correlation was observed between the last two parameters (**Figure 4G**). Following treatment with CXCL9(74-103), infected mice did not show body weight loss until day 4 after infection, with a significant reduction only at day 5, albeit less intense than the vehicle group (**Figure 4B**). In addition, this treatment prevented the increase of CXCL10 expression and the high clinical score (**Figures 4D, E**, respectively). Moreover, the peptide was able to reduce lung viral loads (**Figure 4F**).

**Figure 5:**
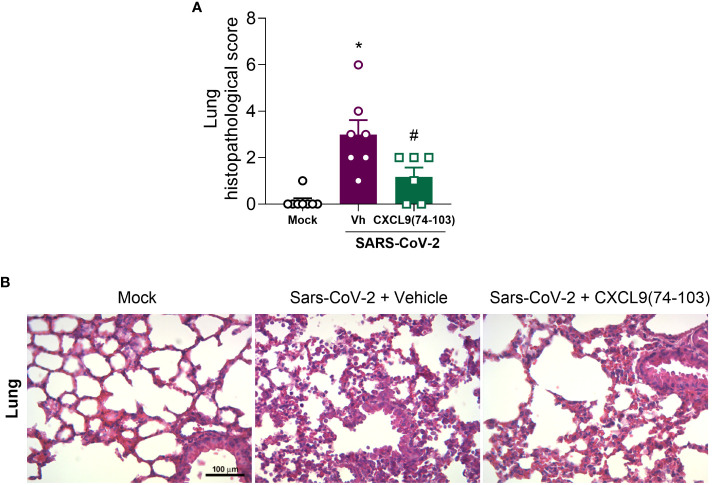
CXCL9(74-103) treatment improves tissue damage in Sars-Cov-2 infection. K18-hACE2-mice were intranasally infected with SARS-CoV-2 (10^5^ PFU/mouse) or DMEM (Mock group) and dissected 5 days later. Mice were intravenously treated twice daily with CXCL9(74-103) since day 0. Vehicle group (-) was infected with SARS-CoV-2 and treated twice daily with 0.9% saline solution. **(A)** Histopathological score in the lung tissue. **(B)** Representative hematoxylin and eosin-stained preparations of lung tissue. Scale bar: 100 μm, as reported in the figure. In panel **(A)**, data are shown as mean ± SEM. Each symbol in panel **(A)** represents data of an individual mouse. *p< 0.05 when compared with the healthy, unchallenged mock group. #p<0.05 when comparing the peptide-treated group with vehicle (Vh).n=6-8.

As the administration of CXCL9(74-103) demonstrated efficacy in reducing clinical manifestations of the disease, we conducted a histopathological analysis of the lungs. As observed in **Figure 5**, the infection with SARS-CoV-2 resulted in an increased histopathological score in the lungs when compared with the mock group. Following treatment with CXCL9(74-103), the histopathological score returned to basal levels (**Figure 5A**). Representative images of the lungs can be observed in **Figure 5B**.

Altogether, just as observed in the MHV-3 model, the administration of CXCL9(74-103) was able to prevent body weight loss, lessen clinical manifestations of the disease, decrease the elevated viral titer in the lungs, and reduce the expression of CXCL10 (**Figure 4**). Notably, this treatment also decreased lung tissue damage, suggesting its potential efficacy in addressing lung pathologies, including COVID-19.

## 4 Discussion

Exploring COVID-19 and different intervention strategies is crucial for elucidating the details of the virus-host interaction and the immune response. In this context, inflammation is recognized to play a pivotal role, and a comprehensive understanding of these dynamics is essential. Learning how the recruitment of cells and the activity of chemokines in general are involved in the pathogenesis of COVID-19 can provide valuable insights into the mechanisms governing the severity and progression of the disease. In addition, investigating the modulation of chemokine functionality may help in the development of potential therapeutic options. Here, we explored the effects of CXCL9(74-103) therapy through two distinct approaches to study SARS-CoV-2 using mouse models. It is important to notice that, for the MHV-3 model, different starting points for treatment were used, giving different outcomes. In most instances, pretreatment was the optimal approach and significantly reduced inflammation and lung complications. However, some experiments, such as the histopathological analysis, showed superior results with the later treatment, demonstrating that the kinetics of treatment and disease is very important and should be further explored and considered when planning future experiments and broader studies. Recognizing that the ideal candidate for a clinical trial should function as a post-infection treatment, there remains room for refining our treatment scheme. In addition, it is known that the lung inflammation caused by MHV-3 is not as extreme as expected for COVID-19 and that this virus has a strong tropism for the liver, but the model presents important disease hallmarks (20).

In the MHV-3 model, the treatment reduced the accumulation of neutrophils in the lungs (**Figure 1C**), together with the improvement of lung dysfunction (**Figure 2**) and lung damage (**Figure 3**). Therefore, the reduction in neutrophil recruitment in this model might be beneficial and should be explored. In coronavirus-induced pneumonia, the number of neutrophils is significantly higher (57–59). Several studies show that excessive neutrophil numbers or neutrophil products are associated with disease severity and tissue damage. For instance, neutrophils in COVID-19 patients have enhanced neutrophil extracellular trap (NET) formation (43, 60) and these NETs have the potential to cause the death of lung epithelial cells (43, 61). Furthermore, the recruitment of neutrophils to the lungs in COVID-19, coupled with the excessive production of reactive oxygen species (ROS), could amplify a local inflammatory response, escalating it to a systemic and more severe level (62). These studies clearly indicate that controlling the massive accumulation of neutrophils and their activated state in lungs of COVID-19 patients are promising strategies to reduce clinical complications of this infection.

Due to the key role of neutrophil-induced lung damage, neutrophils have emerged as a therapy target not only for COVID-19, but for several lung diseases associated with excessive inflammation leading to tissue damage (63–67). Baricitinib, a JAK1/JAK2 inhibitor, caused a reduction in lung infiltration by inflammatory cells, including neutrophils, and, consequently, controlled lung pathology in a model of COVID-19 (68). Similarly, neutrophil-predominant immune responses are associated with worse outcomes in influenza infections (69). Reparixin, a CXCL8 inhibitor targeting its two receptors CXCR1/2, has been tested and showed a promising trend towards limiting disease progression (70). Additionally, CXCR2 inhibitors, such as Navarixin, have also been suggested as a treatment for COVID-19 (71). Broad-spectrum anti-inflammatory medications, such as dexamethasone and methylprednisolone, have also been explored as potential treatments for COVID-19. While these drugs have shown promising results in reducing mortality rates (72, 73), it is important to acknowledge the associated risks. Patients receiving corticosteroid treatment may experience various side effects and have an elevated risk of post-treatment infections (73). Moreover, corticosteroids can disrupt normal organ function and lead to numerous clinical manifestations, potentially exacerbating the risk and severity of COVID-19 complications (74). Therefore, the development and utilization of more targeted and novel treatments that specifically modulate neutrophil recruitment without affecting the entire inflammatory response is very relevant. Such treatments could offer a more precise and effective approach to managing COVID-19 infection while mitigating the adverse effects associated with broad-spectrum anti-inflammatory drugs. Furthermore, the COVID-19 pandemic highlighted the limitations of currently available drugs in effectively managing all forms of inflammation. Encouraging and supporting the advancement of novel therapeutic agents is necessary to address the diverse and evolving challenges posed by inflammatory conditions, including those associated with COVID-19.

In the SARS-CoV-2 model, the administration of CXCL9(74–103) improved clinical manifestations of the disease (**Figure 4E**), as well as reduced the lung tissue damage (**Figure 5**). Despite not affecting the number of blood leukocytes (**Figure 4C**), the systemic efficacy of CXCL9(74-103) treatment is marked, as it demonstrated the ability to prevent body weight loss (**Figure 4B**). This outcome is likely associated with an improvement in the clinical score. Due to the complex environment of a BSL-3 facility, it is currently not possible to do a more in-depth exploration of the mechanisms responsible for these effects. Given that neutrophils play a crucial role in tissue damage and there is a consistent association between CXCL9(74-103) and the reduction in neutrophil recruitment (45–47, 75), we propose that this association might be relevant in the SARS-CoV-2 context as well. Furthermore, the prevention of an increased expression of CXCL10 after treatment (**Figure 4D**), can also contribute to disease moderation by the chemokine-derived peptide. CXCL10, an inflammatory chemokine, is a chemoattractant for different immune cells, including T cells, dendritic cells, and NK cells, playing a crucial role in combating pathogens (30). In viral infections, CXCL10 contributes to lymphocyte activation, facilitates migration, and promotes the infiltration of specific T cell and NK cell subsets to infection sites (76). Moreover, CXCL10 is a key factor in respiratory syndromes. Elevated levels are observed in the plasma and BALF of COVID-19 patients, showing a correlation with disease severity (77, 78). Similarly, increased CXCL10 levels are reported in patients with SARS or Middle East respiratory syndrome (MERS) (27, 79–81). This elevation has been associated with the lymphopenia observed in COVID-19 (82). Therefore, the modulation of chemokine activity by the GAG-binding peptide CXCL9(74-103) might change the expression of CXCL10, leading to the reduction of inflammation and, consequently, a reduction of lung damage.

Interestingly, the administration of CXCL9(74-103) was able to reduce the inflammation without triggering uncontrolled viral replication in both models (**Figures 1G**, **4F**). Surprisingly, for the SARS-CoV-2 infection, the treatment could even reduce the pulmonary viral load. Although we did not investigate basic mechanisms for a possible anti-viral effect of CXCL9(74-103), focusing only in its pathogenesis, it is known that GAGs play a key role in the initial interaction between viruses and host cells, facilitating viral cell infection. In this context, the application of GAG-binding peptides is effective in reducing viral infections caused by dengue virus, herpes simplex virus (HSV)-1, and respiratory syncytial virus (RSV) (83). Remarkably, heparan sulfate, a GAG expressed in diverse cells, is an essential co-factor for SARS-CoV-2 infection. According to Clausen et al. (84), heparan sulfate engages with the receptor-binding domain of the SARS-CoV-2 spike glycoprotein, positioned adjacent to ACE2. This interaction induces a conformational change in the spike structure, facilitating the binding to ACE2. Given that CXCL9(74-103) can actively bind to heparan sulfate and other GAGs (47, 48), it might act as a limiting factor for SARS-CoV-2 infection, resulting in a decrease in viral titer in the lung tissue. Further experiments should be conducted to thoroughly investigate and substantiate this novel aspect of the peptide. Alternative administration routes, such as delivery pumps, could be used to enhance the treatment scheme for this peptide, improving its bioavailability and the therapeutic regimen.

We acknowledge that CXCL9(74-103) is not yet ready to be considered as an alternative treatment for COVID-19. However, this study demonstrates the potential of this group of drugs and raises important points that require optimization and further exploration. Additionally, our findings confirm the significance of GAGs in infection and inflammation, highlighting the beneficial effects of reducing neutrophil recruitment in COVID-19-like diseases. Furthermore, our study has some limitations, including the restricted number of experiments with SARS-CoV-2 due to constraints related to mouse availability and safety considerations. Additionally, there are inherent differences between our animal models and the human disease. It is important to emphasize that this is a preliminary study, and additional experiments are needed to better elucidate the mechanisms underlying the actions of CXCL9(74-103) and to optimize the peptide for potential therapeutic use. In conclusion, the present study contributes to a better understanding of the disease pathogenesis regarding the role of leukocytes in the tissue damage and paved the way for the development of new therapeutic options not only for COVID-19 but also for other inflammatory diseases and potential future pandemics. The complexity of lung diseases, particularly COVID-19, and the chemokine system implies the necessity of more studies linking the two fields and exploring their particularities.

## Data availability statement

The raw data supporting the conclusions of this article will be made available by the authors, without undue reservation.

## Ethics statement

The animal study was approved by Animal Ethics Committees of UFMG (License 420/2018 and 005/2021). The study was conducted in accordance with the local legislation and institutional requirements.

## Author contributions

VO: Conceptualization, Data curation, Formal analysis, Investigation, Methodology, Validation, Visualization, Writing – original draft, Writing – review & editing. CQ-J: Formal analysis, Investigation, Methodology, Writing – review & editing. DH: Formal Analysis, Investigation, Methodology, Writing – review & editing. FS: Methodology, Writing – review & editing. IC: Methodology, Writing – review & editing. MT: Conceptualization, Funding acquisition, Resources, Supervision, Writing – review & editing. RR: Formal analysis, Investigation, Methodology, Writing – review & editing. PP: Conceptualization, Funding acquisition, Investigation, Project administration, Resources, Supervision, Writing – review & editing. VC: Conceptualization, Funding acquisition, Investigation, Resources, Writing – review & editing. SS: Conceptualization, Funding acquisition, Investigation, Project administration, Resources, Supervision, Visualization, Writing – review & editing. FA: Conceptualization, Funding acquisition, Investigation, Project administration, Resources, Supervision, Visualization, Writing – review & editing.
